# Intestinal stem cells acquire premature senescence and senescence associated secretory phenotype concurrent with persistent DNA damage after heavy ion radiation in mice

**DOI:** 10.18632/aging.102043

**Published:** 2019-06-25

**Authors:** Santosh Kumar, Shubhankar Suman, Albert J Fornace, Kamal Datta

**Affiliations:** 1 Department of Biochemistry and Molecular & Cellular Biology and Lombardi Comprehensive Cancer Center, Georgetown University, Washington, DC 20057, USA

**Keywords:** heavy ion radiation, space radiation, radiotherapy, intestinal stem cell, senescence, premature aging, SASP

## Abstract

Heavy ion radiation, prevalent in outer space and relevant for radiotherapy, is densely ionizing and poses risk to stem cells that are key to intestinal homeostasis. Currently, the molecular spectrum of heavy ion radiation-induced perturbations in intestinal stem cells (ISCs), that could trigger intestinal pathologies, remains largely unexplored. The Lgr5-EGFP-IRES-creERT mice were exposed to 50 cGy of iron radiation. Mice were euthanized 60 d after exposure and ISCs were sorted using fluorescence activated cell sorting. Reactive oxygen species (ROS) and mitochondrial superoxide were measured using fluorescent probes. Since DNA damage is linked to senescence and senescent cells acquire senescence-associated secretory phenotype (SASP), we stained ISCs for both senescence markers p16, p21, and p19 as well as SASP markers IL6, IL8, and VEGF. Due to potential positive effects of SASP on proliferation, we also stained for PCNA. Data show increased ROS and ongoing DNA damage, by staining for γH2AX, and 53BP1, along with accumulation of senescence markers. Results also showed increased SASP markers in senescent cells. Collectively, our data suggest that heavy-ion-induced chronic stress and ongoing DNA damage is promoting SASP in a fraction of the ISCs, which has implications for gastrointestinal function, inflammation, and carcinogenesis in astronauts and patients.

## Introduction

Heavy ion radiation is a major concern in outer space and represents an appreciable dose equivalent of the galactic cosmic radiation (GCR) [1–3]. Heavy ions are also a component of the radiation emitted from the sun during solar particle events (SPEs). Astronauts traveling beyond low earth orbits (LEO) and the protective magnetosphere on long duration space missions such as mission to Mars will be exposed to substantial heavy ion radiation [4,5]. Cumulative doses of heavy ion radiation from such missions remain a major concern for astronauts’ health. Although space radiation is primarily high-energy protons, energetic heavy ions due to their high linear energy transfer (high-LET) per unit volume of tissue traversed are considered most harmful for the astronauts. Mathematical modeling approaches indicate that during a Mars mission ~30% of the cells in astronauts will be traversed by either primary or secondary tracts generated by heavy ion radiation [1–3]. It is predicted that astronauts on an approximately 850 to 1000 days of round trip to Mars will receive 0.30 to 0.42 Gy of space radiation. Considering that ~15% of the GCR is heavy ions, astronauts would be expected to receive 0.05 to 0.07 Gy of heavy ion radiation during a Mars mission [6–8]. However, >40% of the dose equivalent in GCR behind 3 g/cm^2^ of aluminum is contributed by heavy ions [9] and contribution of heavy ions to the GCR dose equivalent will be potentially higher during activities outside the spacecraft shielding. Furthermore, current spacecraft shielding technology is unable to block incoming energetic heavy ion radiation rather heavy ions are expected to generate additional harmful secondary radiation from traversed materials. Currently, uncertainty remains about the long-term effects of low dose heavy ion radiation on intestinal stem cells (ISCs), which are important for maintaining astronauts’ gastrointestinal (GI) health during long duration space missions.

Increasing interest in using heavy ion radiation for treating cancers resistant to conventional photon-based radiotherapy [10–12] is also increasing the risk of heavy ion radiation exposure to ISCs. Although heavy ion radiotherapy is more precise relative to photon-based radiotherapy, normal tissue exposure cannot be completely eliminated especially at the entrance plateau region of the Bragg curve [11,13,14]. While normal tissue toxicity at the entrance plateau region is expected to be low-energy and low-dose, normal tissue in the vicinity of the targeted tumor volume is expected to be exposed to high-dose heavy ion radiation [11,13,14]. Considering that heavy ions have higher relative biological effectiveness (RBE) than photon radiation, they are of particular concern for risk of long-term complications such as second cancers in heavy ion radiotherapy patients. Therefore, elucidating tissue-specific biological responses associated with heavy ion radiation is important for understanding the risk and improving the safety of astronauts as well as radiotherapy patients. We have previously demonstrated persistent oxidative, inflammatory, and metabolic stress in intestinal epithelial cells of C57BL/6J mice up to one year after exposure to heavy ion iron radiation [4,15–18]. We have also reported increased intestinal tumorigenesis in adenomatous polyposis coli (APC) mutant mice after exposure to different types and doses of heavy ion radiation [5,19–21]. Currently, delayed effects of heavy ion radiation on intestinal stem cells (ISCs), which are key to intestinal homeostasis as well as tumorigenesis, are not known due to paucity of sufficient *in vivo* human or animal data. Since there are limitations in obtaining human data due to statistically small number of subjects, animal studies could provide key data required to understand risk to ISCs from heavy ion radiation exposures.

The ISCs play important roles in the renewal of the intestinal epithelial lining through regulated proliferation and differentiation of Lgr5^+^ ISCs residing at the crypt base and Lgr5^+^ ISCs have been reported to be essential for epithelial regeneration after radiation damage [22]. Radiation-induced DNA damage triggers the DNA damage response (DDR) and while higher doses of radiation initiate apoptotic response due to higher damage, lower doses primarily induce cell cycle arrest that could lead to cellular senescence [23,24]. In the activation of DNA damage-induced cell cycle arrest, p21 plays a crucial role by inhibiting CDK2 kinase activity and blocking cell cycle progression [23,25]. However, p21 also drives cellular senescence and overexpression of p21 via p53-dependent and -independent mechanisms has been reported to upregulate senescence genes and downregulates proliferative genes in senescent cells [25]. While p21 is known to play key roles in senescence initiation, p16, a member of the inhibitor of cyclin dependent kinase 4 (INK4) family, is primarily involved in maintaining senescence through elevated expression after DNA damage [23,25]. While increased p16 accelerates cellular senescence, which is considered a safe guard mechanism against carcinogenesis, reports in literature also demonstrate upregulation of p16 in a number of cancers and increased p16 was associated with poor prognosis [25]. Additionally, p19, another member of the INK4 family, has also been linked to DNA damage-induced cellular senescence [23,25]. While nuclear localization of these senescence markers is key to their Cdk-inhibitory roles, cytoplasmic localization of p21, p16, and p19 has also been reported [26–28]. Interestingly, cytoplasmic localization of p21 has been proposed to play an antiapoptotic role through inhibition of apoptosis signal-regulating kinase 1 (Ask1) [27]. Furthermore, cytoplasmic localization of p16 as well as of p19 has also been reported in various cells including in cancer cells with diminished apoptosis [26,28]. Overall, increased expression and cytoplasmic localization of these three proteins is predicted to provide a survival advantage and is consistent with apoptosis resistant phenotype of senescent cells [26–29]. A recent study by Wagner et al. [30], has demonstrated that galactosidase beta 1 (Glb1), which is a lysosomal enzyme and is linked to senescence associated-β-galactosidase (SA- β-gal) activity, is an effective marker of cellular senescence in formalin-fixed paraffin embedded tissues. While the the role of cellular senescence in tumor suppression is well established, it has also been implicated in cancer initiation and promotion because senescent cells are resistant to apoptosis, metabolically active, and could potentially acquire secretory phenotype to secret a host of inflammatory and growth stimulatory factors [23,25]. Since senescent cells remain in position for a long time, acquisition of secretory phenotype known as senescence-associated secretory phenotype (SASP) by some of the senescent cells is expected to tilt the homeostatic balance in tissue microenvironment and in surrounding non-senescent cells towards a chronic disease state [31]. Indeed, our previous study has demonstrated long-term decreased intestinal epithelial cell migration after low-dose heavy ion iron radiation and decreased cell migration was associated with increased SASP signaling [4]. The proposed mechanistic model from our study suggests that heavy ion radiation-induced sub-lethal genotoxic stress is stochastically inducing senescence in a proportion of the crypt cells and some of the senescent cells are acquiring secretory phenotype triggering perturbations of molecular events such as cytoskeletal remodeling involved in coordinated epithelial cell migration in intestine [4].

Although ISCs are key to intestinal epithelial cell migration [32] and high dose γ-rays/x-rays-induced DNA damage has been reported to trigger apoptosis and subsequent loss of Lgr5^+^ ISCs [33], we know very little about the long-term effects of low dose heavy ion radiation on ISC senescence and SASP that have implications for intestinal homeostasis. Here we report that exposure to 50 cGy of iron radiation led to increased reactive oxygen species (ROS), oxidative DNA damage, and DNA double stand breaks (DSBs) 60 d after radiation exposure. We also show that iron radiation-induced DNA damage was associated with stem cell senescence and at least some of senescent stem cells show SASP even 60 d after radiation suggesting long-term effects. Our findings suggest that low doses of heavy ions to normal tissues expected during long duration space flights as well as during radiotherapy could impact ISCs and their niche through acquisition of radiation-induced SASP.

## RESULTS

### Increased ROS in ISCs after heavy ion iron radiation

**Figure 1:**
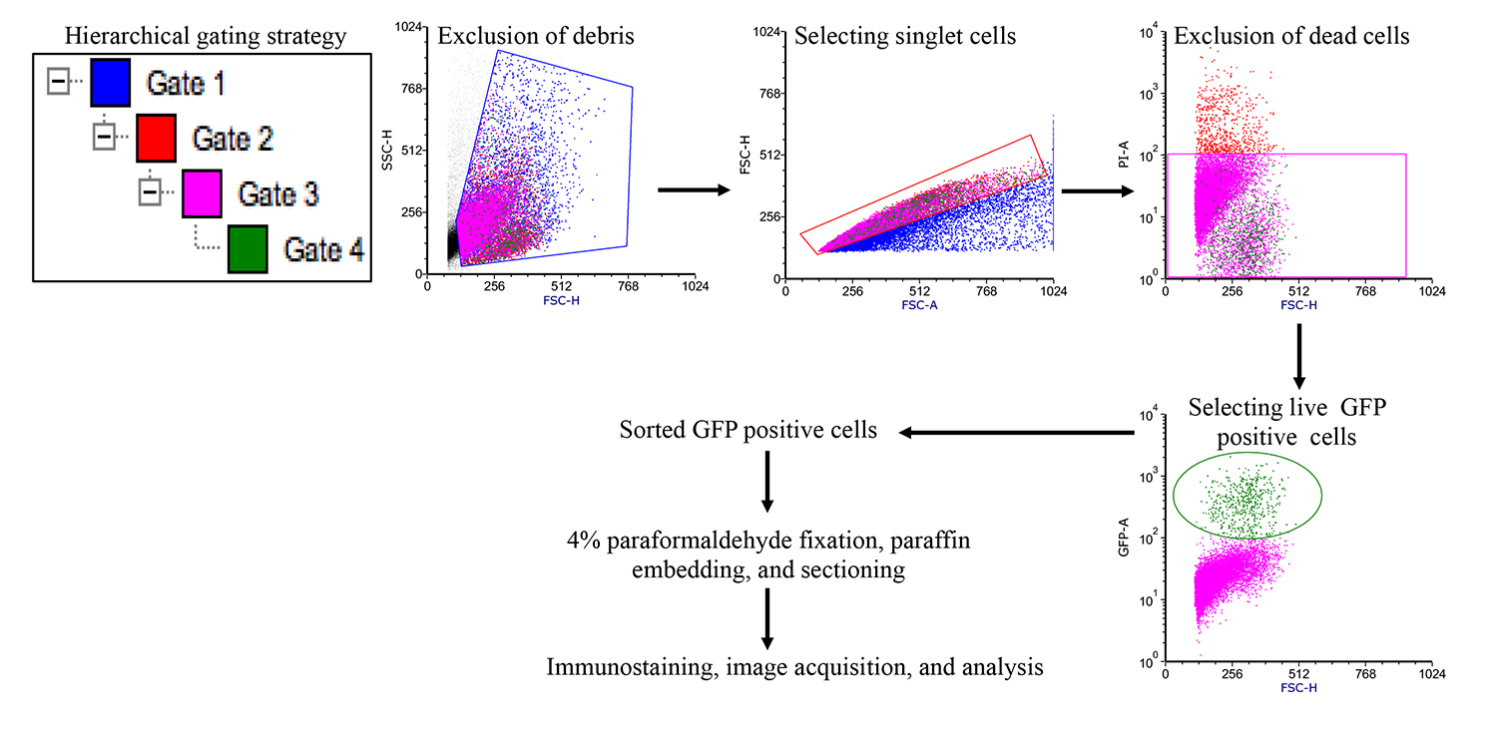
**Fluorescence activated cell sorting strategy to acquire EGFP expressing Lgr5^+^ ISCs.** Initially gates were applied using side scatter and forward scatter parameters to exclude debris and doublet cells. Subsequent gating allowed removal of dead cells and acquisition of EGFP positive ISCs for further processing.

**Figure 2:**
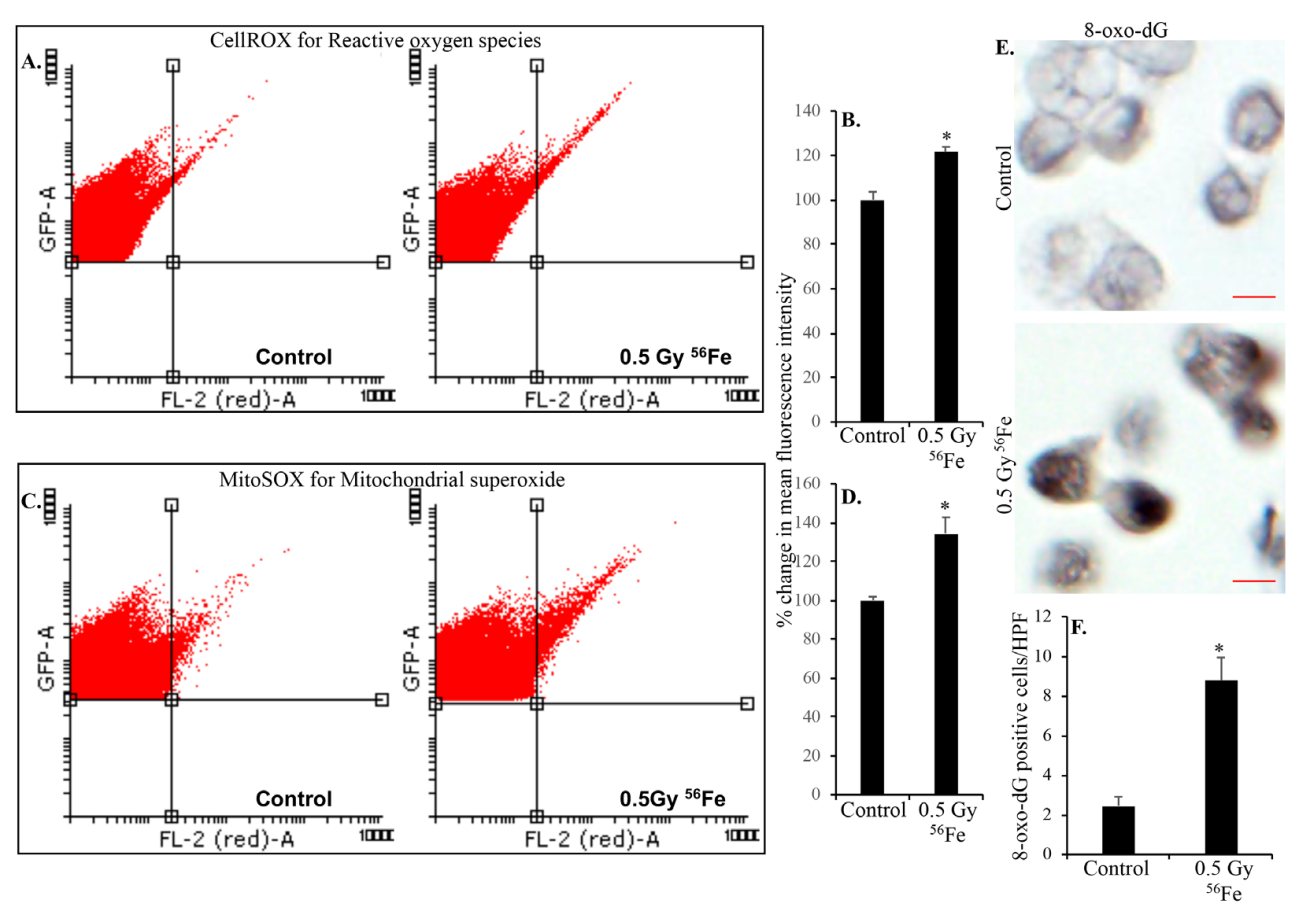
**Heavy ion iron radiation leads to increased ROS and oxidative DNA damage in ISCs two months after exposure.** (**A**) Representative flow cytometry histograms showing increased CellROX fluorescence in the upper right quadrant indicating increased ROS after iron radiation. (**B**) Quantification of mean fluorescent intensity data from five mice are presented as percent change in mean fluorescence in irradiated samples relative to controls demonstrates increased ROS in ISCs of iron irradiated mice. (**C**) Cells were incubated with mitochondrial fluorescent probe MitoSOX Red to assess mitochondrial ROS and representative flow cytometry histograms are presented to show increased mitochondrial ROS after iron irradiation. (**D**) Mean fluorescent intensity data from five mice was are graphically presented as percent change in irradiated relative to control samples. Significantly higher levels of mitochondrial ROS were detected after iron irradiation relative to control. (**E**) Sorted, fixed, paraffin embedded, and sectioned ISCs were stained for 8-oxo-dG and representative immunohistochemistry images are presented showing increased 8-oxo-dG stained nuclei after iron irradiation. Scale bars, 5 μm. (**F**) Quantification of number of 8-oxo-dG positive cells in ISC sections from control and irradiated mice. Data are presented graphically showing significantly higher 8-oxo-dG staining in irradiated samples relative to controls. Error bars show SEM.

Radiation exposure is linked to increased ROS production and oxidative stress and our previous studies have demonstrated chronic oxidative and inflammatory stress in intestinal epithelial cells (IECs) after heavy ion radiation [15]. Since ISCs proliferate and differentiate into IECs, we wanted to test if ISCs themselves have increased ROS levels after heavy ion radiation. Fluorescence activated cell sorting was used to sort EGFP expressing Lgr^+^ ISCs from control and heavy ion iron irradiated mice (Figure 1). Intracellular ROS was assessed in ISCs using fluorescent probe CellROX and flow cytometry. Our data show increased ROS in ISCs indicated by right shift in histogram two months after heavy ion ^56^Fe radiation (Figure 2A). Quantification of the flow cytometry data show significantly higher ROS in ISCs after ^56^Fe radiation relative to control (Figure 2B). Since mitochondria is a major source of ROS, we used mitochondrial superoxide detecting fluorescent probe MitoSOX Red to further assess oxidative stress in ISCs. Increased mitochondrial superoxide (Figure 2C) observed two months after heavy ion radiation and quantification show significantly increased mitochondrial superoxide relative to control (Figure 2D) suggesting perturbed mitochondria in ISCs.

### Heavy ion iron radiation-induced DNA damage and cell proliferation

**Figure 3:**
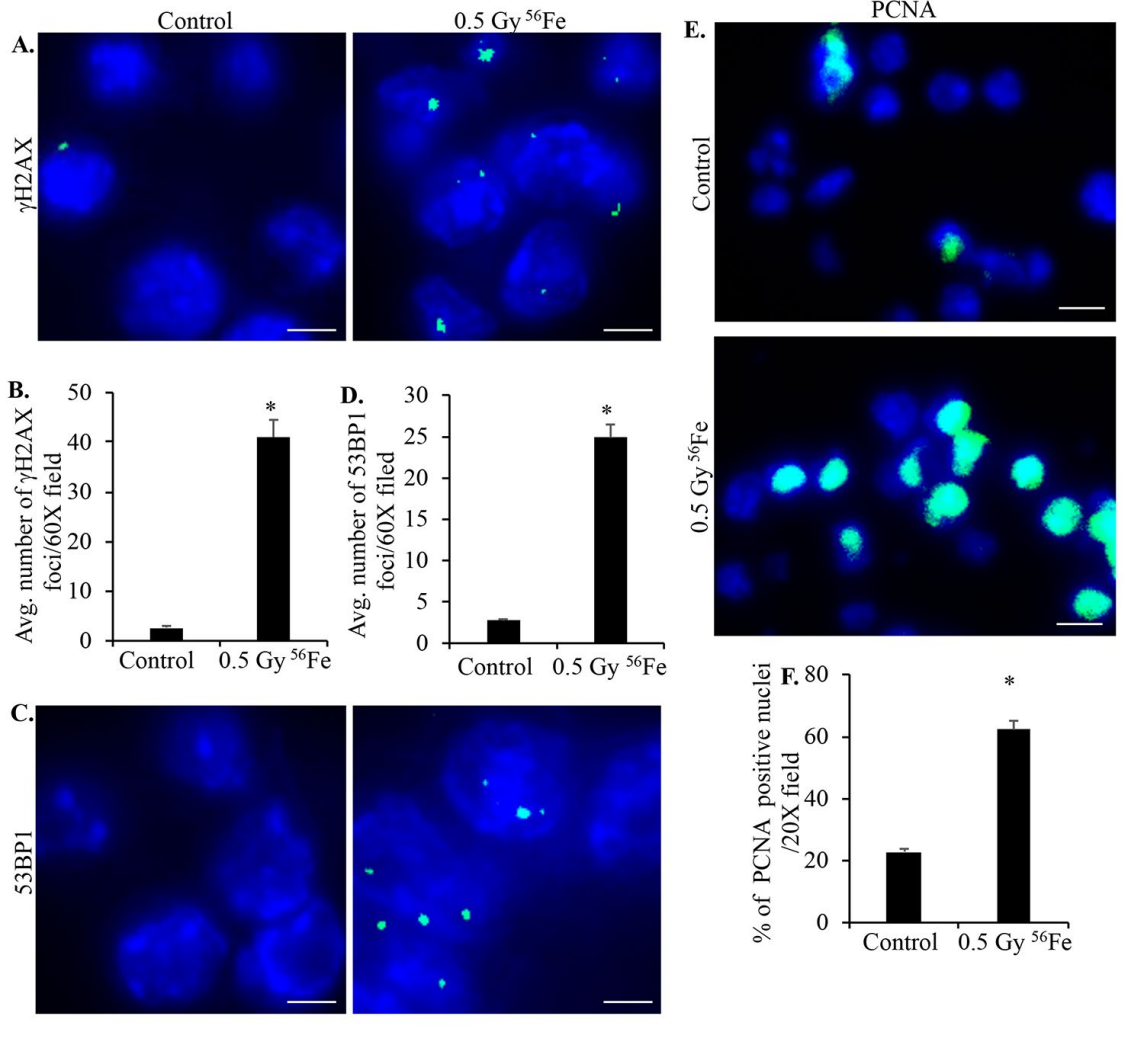
**Murine ISCs show increased DNA DSB and cell proliferation after heavy ion radiation exposure.** (**A**) Representative immunofluorescence (IF) images of murine ISC sections from control and irradiated mice showing increased γH2AX foci in nuclei after iron irradiation. Scale bars, 5 μm. (**B**) Number of γH2AX foci in ISC nuclei from control and irradiated mice were counted and data from five mice presented graphically showing significantly increased foci in irradiated samples. (**C**) Representative IF images showing 53BP1 foci in ISC sections from control and irradiated samples. Scale bars, 5 μm. (**D**) Quantification of 53BP1 foci in control and irradiated sections presented graphically showing increased number of foci in iron irradiated samples. (**E**) Representative IF images of ISC sections stained for PCNA showing increased staining in irradiated relative to control samples. Scale bars, 10 μm. (**F**) Graphical presentation of percent of PCNA positive nuclei in control and irradiated samples from five mice. Error bars show SEM.

Sorted ISCs from control and iron irradiated mice were fixed, embedded, and sectioned for immunostaining. Anti-8-oxo-dG antibody was used to stain ISC sections to assess heavy ion radiation-induced oxidative DNA damage. Increased 8-oxo-dG staining in ISCs is evident after iron irradiation relative to control (Figure 2E and F). Increased ROS is also known to induce DNA strand breaks and since DNA DSBs are the most deleterious form of DNA damage, we used γH2AX and 53BP1 staining to assess DNA DSBs in ISC sections 2-month after iron irradiation. Increased γH2AX (Figure 3A and B) as well as 53BP1 (Figure 3C and D) foci in iron irradiated samples indicate continued presence of DNA DSBs in the ISCs after heavy ion irradiation. Considering that oxidative stress and DNA damages are present in live sorted ISCs and ROS below apoptotic threshold are known to trigger cell proliferation and survival [34], we wanted to test if ISCs are proliferating. We stained ISC sections for PCNA, a known G1/S phase marker [35–37], and show increased PCNA staining in iron irradiated samples (Figure 3E and F).

### Increased senescence and senescence associated secretory phenotype (SASP) markers in ISCs after heavy ion iron irradiation

**Figure 4:**
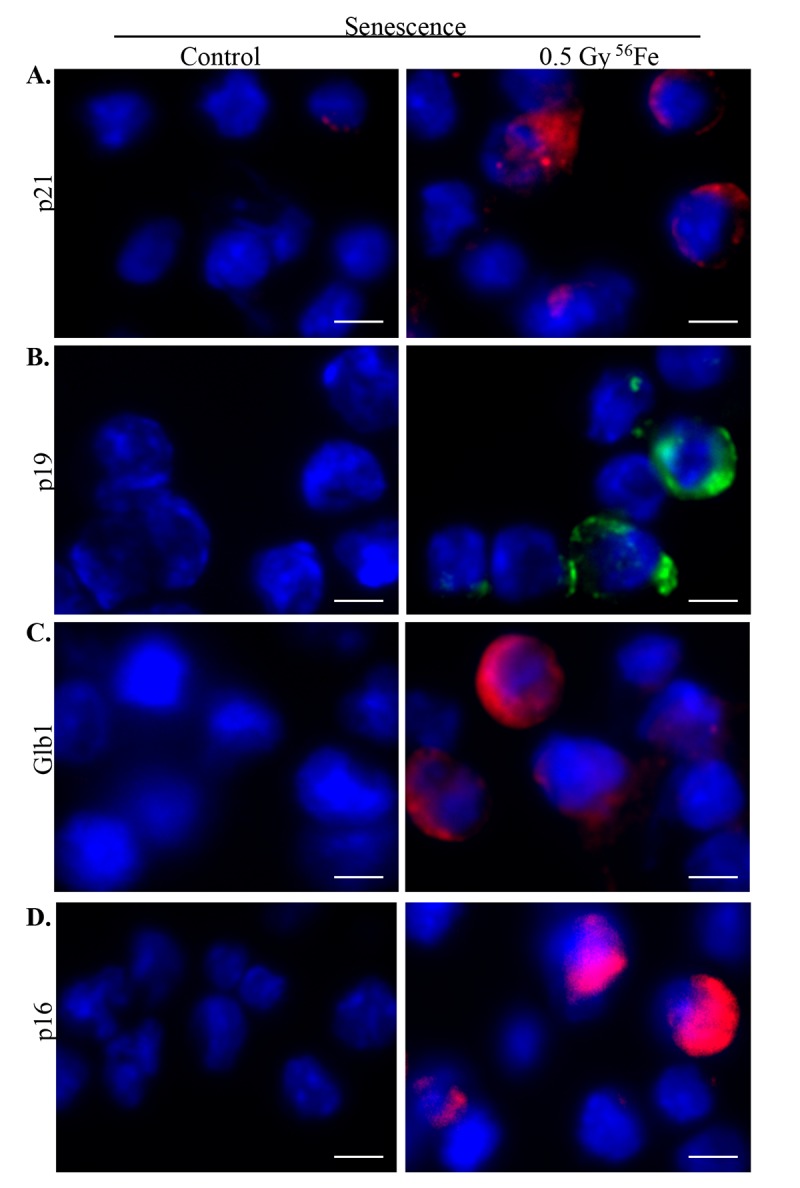
**Increased senescence in murine ISCs after heavy ion radiation exposure.** (**A**) Representative IF images showing increased p21 expression in ISCs from heavy ion radiation exposed mice. (**B**) Increased p19 expression in ISCs from heavy ion radiation exposed mice. (**C**) ISC sections immunofluorescently stained for Glb1 show increased staining in irradiated samples. (**D**) Representative IF images showing increased p16 levels in ISCs from heavy ion radiation exposed mice. Scale bar, 5 μm.

**Figure 5:**
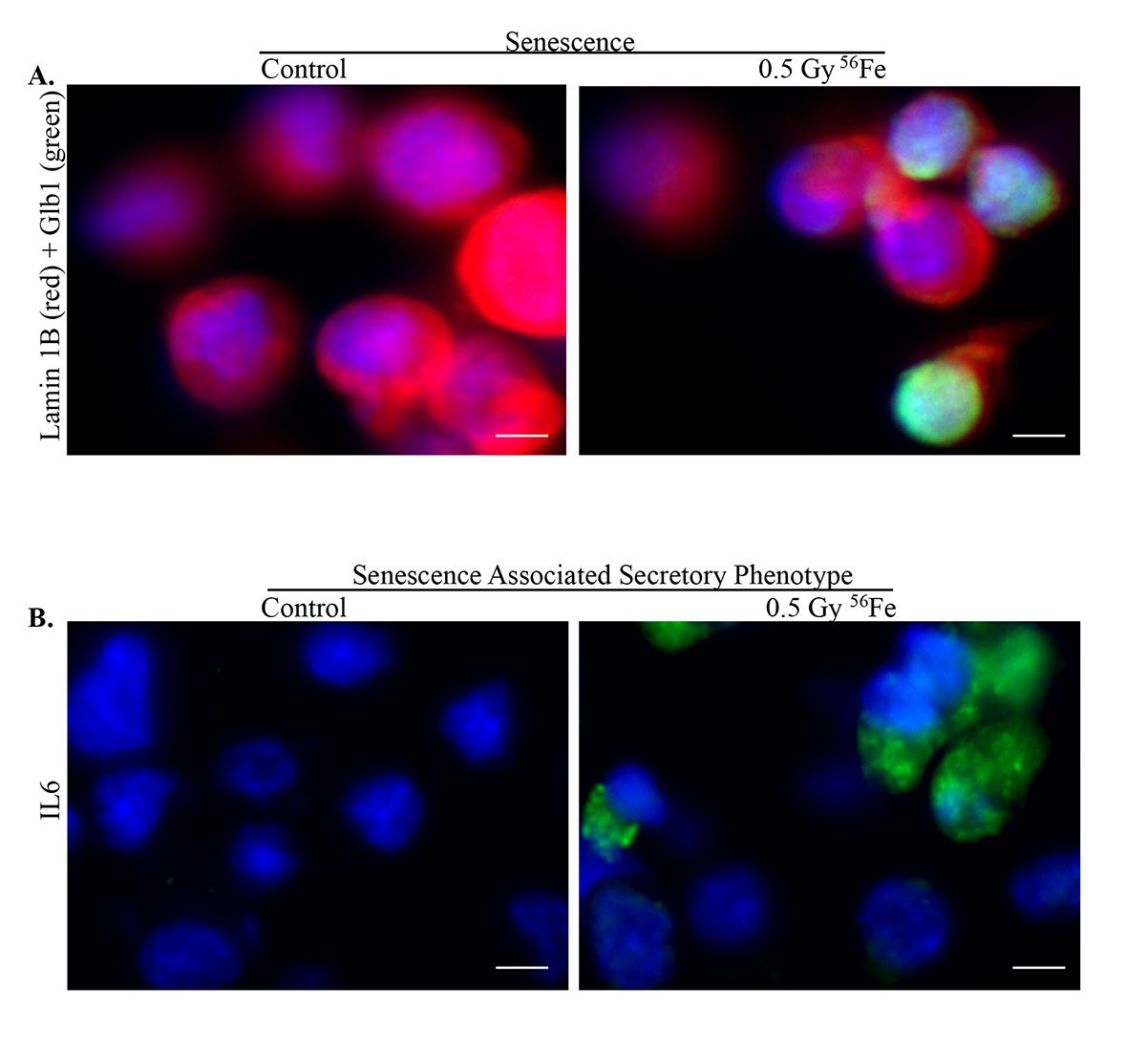
**Senescence and SASP in ISCs after exposure to heavy ion radiation.** (**A**) Representative IF images of ISCs co-stained for senescent markers Lamin 1B (red) and Glb1 (green) showing decreased expression of Lamin 1B in senescent cells. (**B**) Increased expression of SASP marker IL6 after heavy ion iron radiation exposure. Scale bars, 5 μm.

**Figure 6:**
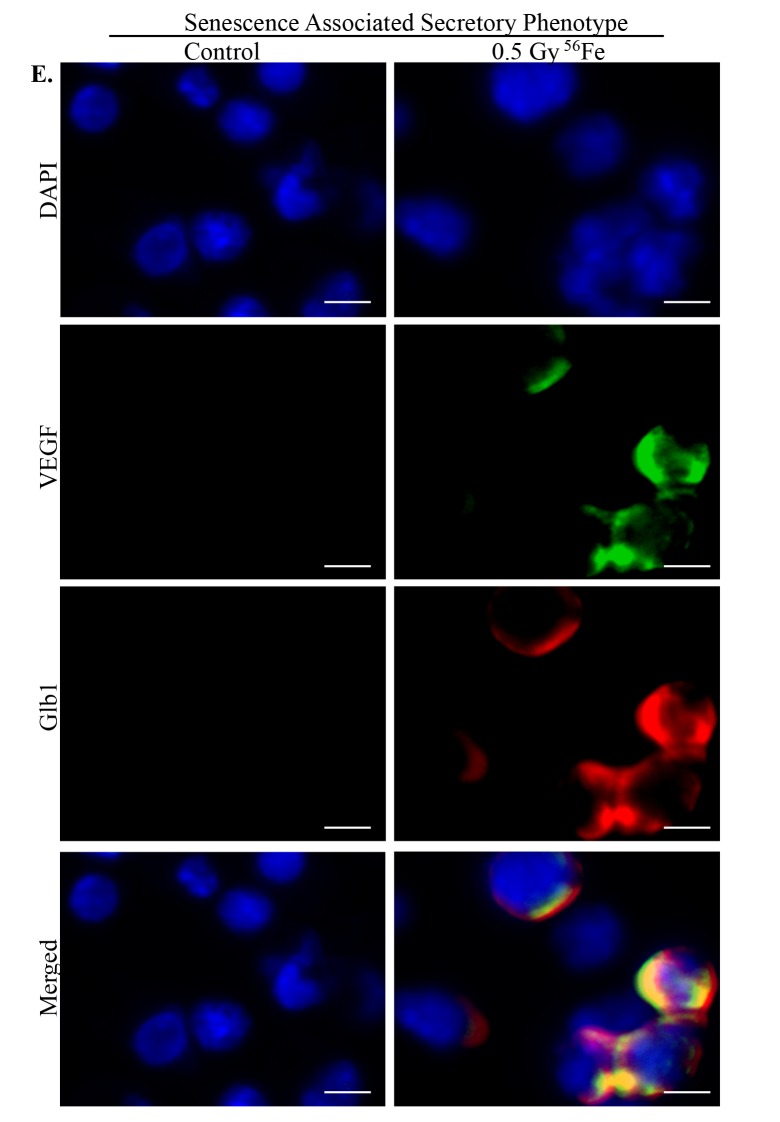
**Acquisition of SASP by heavy ion radiation-induced senescent ISCs.** ISC sections from control and irradiated mice were co-stained for SASP marker VEGF (green) and senescent marker Glb1 (red) showing acquisition of secretory phenotype by senescent cells after heavy ion radiation exposure. Scale bar, 5 μm.

**Figure 7:**
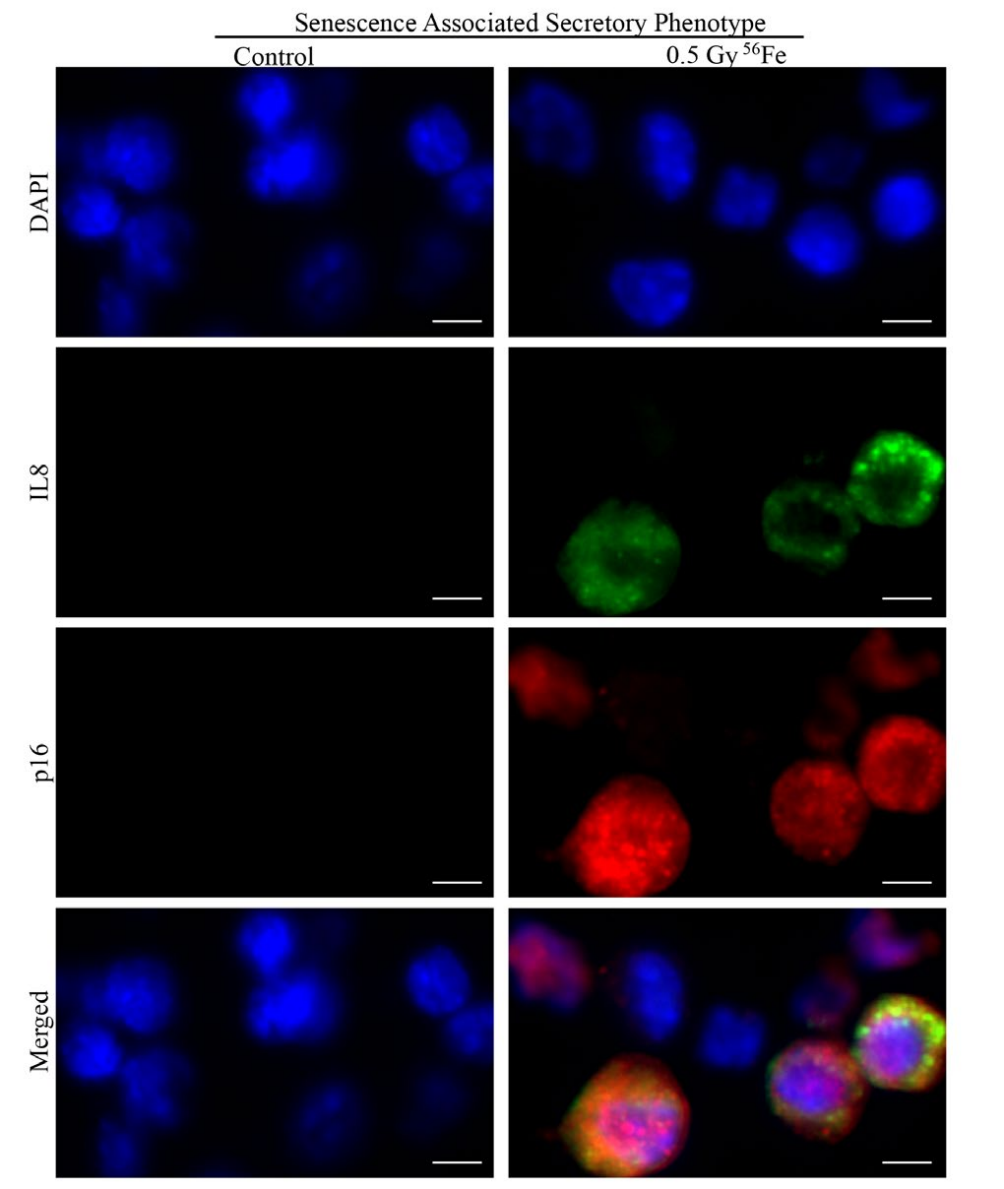
Representative IF images of ISC sections co-stained for SASP marker IL8 (green) and senescent marker p16 (red) showing some of the senescent cells acquiring secretory phenotype in irradiated samples. Scale bar, 5 μm.

Chronic stress and DNA damage is linked to cellular senescence [4,38,39] and indeed, we observed increased expression of multiple markers of senescence two months after heavy ion iron radiation. Here, we report upregulation of p21 (Figure 4A), p19 (Figure 4B), Glb1 (Figure 4C) and p16 (Figure 4D) in iron irradiated samples. We observed diffuse cytoplasmic and nuclear staining for p21, p19, Gbl1, and p16 and is consistent with previously reported staining pattern for these markers of cellular senescence [30,40–44]. We also show decreased expression of Lamin 1B (Figure 5A) in Glb1 stained senescent ISCs relative to non-senescent ISCs further supporting cellular senescence. Cellular senescence is invariably associated with some of the senescent cells acquiring SASP essentially secreting pro-inflammatory and pro-proliferative factors and thus affecting local tissue microenvironment. Our co-staining data show increased levels of known SASP markers IL6 (Figure 5B), VGEF (Figure 6), and IL8 (Figure 7) in some of the senescent cells two months after exposure to heavy ion iron radiation. Since secretory senescent cells are implicated in perturbations of tissue microenvironment as well as of non-senescent cells, we counted number of p16 (senescent) vs. p16+IL8 (SASP) cells per field of vision (FOV) in iron radiation samples. The quantification data show that 44% of the senescent cells acquired secretory phenotype (SASP) after heavy ion irradiation.

## DISCUSSION

In this study, we used a ‘knock-in’ mouse model [45], which expresses EGFP and CreERT2 replacing Lgr5 expression in Lgr5^+^ stem cells (Lgr5-EGFP-IRES-creERT mice) to define long-term *in vivo* effects of heavy ion iron radiation on ISCs. Data show heavy ion radiation induced oxidative stress in ISCs via increased production of ROS including mitochondrial ROS. Since oxidative stress and accompanied increased oxidative DNA damage and DNA DSB were observed in these live sorted ISCs, the current data suggest that levels of cellular stress and DNA damage in these cells were below apoptotic threshold. Sub-lethal ROS is known to trigger cell proliferation and indeed, data show increased PCNA staining in iron irradiated samples suggesting increased entry of ISCs into the cell cycle (G1/S). Since PCNA is a G1/S marker [35–37] and we noted DNA damage in a subset of ISCs, it is possible that some of the DNA damage containing cells are entering cell cycle. However, because our data show ISC senescence, we believe some of the ISCs with DNA damage have also entered senescence and therefore, these are excluded from the possibility of cell cycle entry. At this time, we cannot dismiss the possibility that a subset of DNA damage containing cells are entering cell cycle to divide and proliferate and another subset of cells with DNA damage in entering senescence rather than continuing with cell division. It is conceivable that there is a DNA damage threshold that determines proliferation vs. senescence in damage bearing ISCs and both these outcomes have the potential for functional alterations and oncogenic transformations in gastrointestinal tissue. The oncogenic transformation potential gained further credence from our observation of heavy ion radiation-induced SASP in ISCs. In summary, data from the current study broadened our understanding about the susceptibility of ISCs to heavy ion radiation and describes a role for SASP in increasing the long-term risk to ISCs and thus intestinal homeostasis in astronauts and patients.

Radiation is known to promote increased oxidant production beyond cellular antioxidant capacities leading to increased DNA damage [15]. Heavy ion radiation due to its high-LET characteristics and dense ionization events in tissues has been reported to elicit persistent stress in different cell types with potential for chronic diseases including cancer and neurodegeneration [4,15,16,18,46–48]. Data from the current study show that heavy ion radiation-induced ROS and DNA damage was detectable two months after exposure suggesting long-term effects on ISCs. Our flow cytometry data showing increased mitochondrial ROS suggest heavy ion radiation-induced mitochondrial perturbation. Emerging evidence suggests that while a lower level of ROS is essential for maintenance of stemness in a variety of adult stem cell types, a higher but non-lethal level of ROS has been reported to promote stem cell proliferation including ISC in *Drosophilla* and mice [49,50]. Importantly, enhanced cell proliferation also has the potential of generating DNA DSB due to replication stress from replication fork collapse [51]. Our data of mitochondrial perturbation, and increased ROS and cell proliferation when considered with evidence in literature suggest that heavy ion radiation is promoting a permissive state of ROS accumulation, DNA damage, and growth factor stimulation concurrently in ISCs [49,52].

Accumulating evidence supports the notion that oxidative stress and accompanying damage to biomolecules including DNA, proteins, and lipids is involved in organismal aging and cellular stress and DNA damage is a major factor contributing to stem cell aging [49,53]. In our model, heavy ion radiation-induced chronic oxidative stress and DNA damage was associated with an increased number of senescent ISCs, and given the age of these mice at the time of tissue harvesting and results in unirradiated cells, the senescent phenotype is premature. Radiation via promotion of oxidative stress and macromolecular damage including damage to DNA is known to usher in premature aging based on epidemiological and animal studies due to ‘stress-induced premature senescence’ [4,54]. Our data suggest that a significant fraction of the ISCs enter into ‘stress-induced premature senescence’ after heavy ion radiation exposure and senescence has been reported to affect stem cell dynamics with implications for chronic diseases including cancer and aging-associated diseases. While senescent cells are resistant to mitogenic and apoptotic signals and irreversibly arrested in G0/G1 phase, they are metabolically active with altered gene and protein expression patterns [55]. A key factor for maintenance of senescent state is continued blocked of cell cycle progression though increased expression of several cyclin-dependent kinase (CDK) inhibitors such as p21, p16, and p19 [49,53,55]. Our study provided evidence that some of the ISCs are expressing these CDK inhibitors, which are considered hallmarks of cellular senescence, and is consistent with our previous report on heavy ion radiation-induced intestinal epithelial cell senescence and SASP affecting epithelial cell migration in intestine [4]. While these CDK inhibitors have both cytoplasmic and nuclear localization, their cytoplasmic localization blocks cell death [26]. Importantly, our data show both cytoplasmic as well as nuclear localization of CDK inhibitors in the sorted ISCs. ISC senescence has been further confirmed by staining for additional recognized markers including Glb1 and Lamin B1. Increased expression of Glb1, which encodes lysosomal-β-galactosidase and is responsible for known senescent marker senescence-associated-β-galactosidase (SA-β-gal) activity, has recently been reported as a marker for senescent cells in fixed paraffin embedded sections [30]. Conversely, Lamin B1, which is expressed in all cell types and is involved in stress responses and cell division, is decreased in senescent cells [56,57]. Our co-staining data with Glb1 demonstrate that Lamin B1 is decreased in senescent ISCs supporting our heavy ion radiation-induced premature aging phenotype hypothesis. Senescence in a fraction rather than all of ISCs could be attributed to energy deposition pattern of heavy ion radiation where part of the energy is deposited in cells by secondary δ-rays far away from the traversing primary particle resulting in non-uniform distribution of damage [58]. Importantly, while assessing risk to astronauts from low dose space radiation, it is also crucial to consider non-targeted or bystander effects where stress responses from cells hit by primary or secondary radiation are propagated to nearby non-hit cells [59]. Hence, heavy ion radiation-induced oxidative stress, DNA damage, and senescence in ISC are likely to involve targeted as well as non-targeted effects.

While senescence through terminal growth arrest acts as a protective step against transformation and tumorigenesis, it also allows cells to survive long-term and release pro-inflammatory and pro-growth molecules affecting tissue microenvironment [55,60]. Acquisition of tissue microenvironment altering SASP has been implicated in chronic diseases including cancer and inflammatory conditions [55,60]. Radiation including proton radiation has been reported to induce senescence and SASP to promote oxidative and inflammatory stress and increase tissue vulnerability to disease processes [61,62]. The current study shows that some senescent ISCs acquired SASP demonstrated by increased pro-inflammatory IL6 and IL8 as well as pro-angiogenic VEGF after heavy ion radiation exposure suggesting a pervasive pro-inflammatory and pro-proliferative stress in the crypt-base stem cells. However, if a proportion of the crypt-base Lgr5^+^ stem cells are senescent and some of the senescent cells acquire SASP, the question remains how proliferative compartment is maintained. It is possible that SASP-induced growth stimulatory stress is extended to the +4 stem cells, which are considered quiescent and relatively radioresistant but are activated by stress, to expand proliferating Lgr5^+^ stem cell compartment [33,63]. Notably, SASP response is amplified through propagation from cell to cell promoting more cellular senescence, inflammation, ROS production, and DNA damage and it is plausible that pharmacological elimination of senescent cells could break this cycle of perpetual stress. We acknowledge that astronauts on a return trip to Mars are estimated to receive 0.30 to 0.42 Gy of space radiation [6–8]. Since approximately 15% of the galactic cosmic radiation (GCR) is contributed by heavy ions [5], it is expected that astronauts will receive about 0.07 Gy (7 cGy) of heavy ions during a Mars exploratory mission. The dose of heavy ion radiation used in the current study, while higher than predicted during Mars missions, provide a glimpse into the long-term intestinal tissue response after heavy ion radiation exposure. Heavy ion radiation is also used in treating cancers resistant to conventional radiotherapy and since heavy ion radiotherapy is of higher doses relative to space travel, our study may contribute to understanding risk of second cancers in patients [11]. Therefore, this initial study provides a foundation for further research on heavy ion radiation-induced changes in ISC morphology, function, and microenvironment that could contribute to understanding the pathogenesis of gastrointestinal disorders in astronauts and radiotherapy patients. Importantly, our data suggest that SASP is a targetable risk factor and that its underlying mechanisms could be modulated to prevent or delay heavy ion radiation-induced premature senescence related GI functional decline and cancer growth.

## MATERIALS AND METHODS

### Mice and radiation

Male Lgr5-EGFP-IRES-creERT mice [45] were purchased from Jackson Laboratory (Stock No: 008875, Bar Harbor, ME, USA) and housed at Georgetown University (GU) animal care facility. Mice (n=10/study group) were exposed to whole body iron (^56^Fe) radiation (energy: 1000 MeV/nucleon; LET: 148 keV/μm; dose: 50 cGy) using the simulated space radiation facility at NASA Space Radiation Laboratory (NSRL) in Brookhaven National Laboratory (BNL), and control mice were sham irradiated. All mice were shipped a week before radiation exposure for acclimatization at the BNL animal facility and returned a day after radiation exposure to the GU animal facility in a temperature-controlled environment with an aim to minimize transportation related stress. Mice were housed in an air- and temperature-controlled room with 12-hour dark and light cycle maintained at 22 °C in 50% humidity at the GU as well as at the BNL animal care facility. All the mice were provided food and filtered water *ad libitum*. All animal procedures were approved by the Institutional Animal Care and Use Committees at BNL (Protocol#345) and GU (Protocol#2016-1129). Our research followed the Guide for the Care and Use of Laboratory Animals, prepared by the Institute of Laboratory Animal Resources, National Research Council, and U.S. National Academy of Sciences.

### Intestinal epithelial cell isolation

Mice were euthanized 60 d after radiation exposure and small intestine was carefully dissected out. After flushing with PBS to remove fecal matter, small intestine from each mouse was cut into smaller pieces (~10 mm), lumen inverted, and total intestinal epithelial cells including ISCs from the crypt area were isolated with a protocol standardized in the laboratory [15] with modifications. Briefly, intestinal sections with inverted lumen was placed in a solution containing 27 mM sodium citrate, 1.5 mM KCl, 96 mM NaCl, 8 mM KH2PO4 and 5.6 mM NaH2PO4 at pH 7.3 for 15 min. Subsequently, intestinal sections were incubated with 2 mM EDTA in PBS for 20 min, then washed vigorously twice with PBS. The intestinal sections were then incubated in a cell dissociation enzyme mix containing Collagenase Type 1 (60 U/ml) and Neutral protease (2.4 U/ml) in HBSS for 20 min at 37 °C. Cell dissociation enzyme mix was discarded, tissues were placed in cold HBSS, and cells were released with vigorous shaking. Released cells were passed through a 70-micron mesh (BD Biosciences, Bedford, MA) to remove clump and debris, and obtain a uniform single cell suspension. Cells were centrifuged, washed twice with cold HBSS, and were resuspended in HBSS containing 2% FBS for fluorescence activated cell sorting of Lgr5^+^ ISCs.

### Fluorescence activated cell sorting (FACS) of EGFP expressing Lgr5^+^ ISCs

Single cell suspension of intestinal epithelial cells was sorted on FACS ARIA IIU (Becton Dickinson, San Jose, CA) sorter using FACSDIVA software. The sorting strategy is schematically presented in Figure 1. Proper electronic gates of side scatter and forward scatter parameters were set to exclude debris and doublet cells. Viable cells were gated using negative SYTOX Blue (Cat#S34857, Life Technologies) staining to exclude dead cells with 405 nm laser through 450/40 band pass filter. EGFP positive cells were sorted and collected with 488 nm laser through a 530/40-band pass filter. While live sorted Lgr5^+^ cells were used to assess intracellular reactive oxygen species (ROS) and mitochondrial superoxide, sorted cells were also fixed in 4% paraformaldehyde, pelleted, paraffin embedded and sectioned for immunostaining.

### Measurement of ROS and mitochondrial superoxide in EGFP expressing Lgr5^+^ ISCs

Intracellular ROS and mitochondrial superoxide were analyzed in ISCs using fluorescent probes CellROX (Cat# C10491, Life Technologies) and MitoSOX Red (cat# M36008 Life technologies) respectively as described previously [15]. Briefly, cells were incubated with either 5 µM CellROX for 30 min or 2 µM MitoSOX Red for 10 min in dark at 37 °C, washed in PBS once, resuspended in 500 μl of PBS, and fluorescence intensity acquired by flow cytometry (LSRFortessa, BD Biosciences, San Jose, CA). Cells were counter-stained with SYTOX™ Blue (cat#S34857, Thermo Fisher Scientific, Waltham, MA) to exclude dead cell while acquiring data. After data acquisition, flow cytometry files (.fcs files) were analyzed using Flowing Software v2 (Cell imaging core, Turku Center for Biotechnology, available at http://flowingsoftware.btk.fi/). Initially, a gate (gate-1) for high FSC and SSC signal in the dot-plot was applied to exclude cell debris followed by a second gating (gate-2) to pick EGFP positive cells that represent the ISCs. The cell population obtained after gate-2 was analyzed for FL1 (EGFP) vs FL2 (CellROX or MitoSOX) population and a quadrant was applied for analysis of any population shift towards the right indicating increased fluorescence. Cells isolated from 5 mice were acquired and analyzed using the same setting and cell population percentage in upper right quadrant representing cells with higher intracellular and mitochondrial ROS is presented graphically as mean ± standard error of the mean (SEM).

### Assessing oxidative DNA damage in ISCs

We used sorted, fixed, embedded and sectioned ISCs for 8-oxo-dG staining, a known marker of oxidative DNA damage. Sections were deparaffinized, sequentially rehydrated, and treated with a primary antibody specific to 8-oxo-dG (Trevigen, Gaithersburg, MD) according to a protocol described previously [15]. Signals were detected using DAB substrate provided in the Mouse and Rabbit Specific HRP/DAB IHC Detection Kit - Micro-polymer (Cat# ab236466, Abcam, Cambridge, MA) according to the manufacturer’s instruction. Sections were counterstained with hematoxylin, sequentially dehydrated, and mounted using Permount mounting medium (Thermo Fisher Scientific). Mounted slides were visualized under a bright field microscope and images were captured. Images were quantified using ImageJ v1.51 and the error bar represents SEM.

### Immunofluorescence

Sections were deparaffinized, sequentially rehydrated, and antigen retrieved in citrate buffer, pH 6.0 (Electron Microscopy Sciences, Hatfield, PA). Samples were incubated overnight with a specific primary antibody for γH2AX (cat# 4418-APC-100; dilution-1:50; Trevigen), 53BP1 (cat#bs3020R; dilution-1:30; Bioss Antibodies Inc., Woburn, MA), p19 (cat#07-543 ; dilution-1:100 ; Millipore, Burlington, MA), IL8 (cat#orb229133; dilution-1:200; Biorbyt LLC, San Francisco CA), IL6 (cat#ab7737; dilution-1:200; Abcam, Cambridge, MA), Ki67 (cat#ab15580; dilution-1:200; Abcam), Glb1 (cat#ab203749; dilution-1:200; Abcam), p16 (cat#sc-1661; dilution-1:200; Santa Cruz Biotechnology, Dallas, TX), Lamin 1B (cat # sc-17810; dilution-1:200; Santa Cruz Biotechnology) VGEF (cat# sc-7269; dilution-1:200; Santa Cruz Biotechnology), or p21 (cat#05-345 ; dilution 1:100 ; Millipore) at 4 °C. Sections were washed and treated with a secondary antibody conjugated with AlexaFluor 488 (green) or 546 (red) for 1 h at room temperature. Nuclei were counter stained with DAPI. Sections were visualized and images captured using an Olympus BX61 DSU microscope at microscopic magnifications indicated in the figures. Images were acquired and processed using cellSens Entry v1.15 (Olympus Corp, Center Valley, PA) for immunohistochemistry, and Slidebook v6 (Intelligent Imaging Innovations, Denver, CO) for immunofluorescence. Ten randomly chosen field of vision (FOV) were captured in each study group and a representative image for each group is presented in the results. Quantification data show average number of positive cells per FOV and the error bar represents SEM. The number of γH2AX and 53BP1 foci in each cell nucleus were counted visually in Slidebook v6 and data presented as average number of foci per 60X field. Statistical significance between the two groups was determined using Student’s t-test and p<0.05 was set for significance. Appropriate controls were run in parallel with the experimental sections to assess specificity of the immunostaining.

## References

[1] Curtis SB, Letaw JR. Galactic cosmic rays and cell-hit frequencies outside the magnetosphere. Adv Space Res. 1989; 9:293–98. 10.1016/0273-1177(89)90452-3 11537306

[2] Hayatsu K, Hareyama M, Kobayashi S, Yamashita N, Sakurai K, Hasebe N. HZE Particle and Neutron Dosages from Cosmic Rays on the Lunar Surface. J Phys Soc Jpn. 2009 (Suppl A); 78:149–52. 10.1143/JPSJS.78SA.149

[3] Setlow RB. The hazards of space travel. EMBO Rep. 2003; 4:1013–16. 10.1038/sj.embor.7400016 14593437 PMC1326386

[4] Kumar S, Suman S, Fornace AJ Jr, Datta K. Space radiation triggers persistent stress response, increases senescent signaling, and decreases cell migration in mouse intestine. Proc Natl Acad Sci USA. 2018; 115:E9832–41. 10.1073/pnas.1807522115 30275302 PMC6196540

[5] Suman S, Kumar S, Moon BH, Strawn SJ, Thakor H, Fan Z, Shay JW, Fornace AJ Jr, Datta K. Relative Biological Effectiveness of Energetic Heavy Ions for Intestinal Tumorigenesis Shows Male Preponderance and Radiation Type and Energy Dependence in APC(1638N/+) Mice. Int J Radiat Oncol Biol Phys. 2016; 95:131–38. 10.1016/j.ijrobp.2015.10.057 26725728

[6] Cucinotta FA, Durante M. Cancer risk from exposure to galactic cosmic rays: implications for space exploration by human beings. Lancet Oncol. 2006; 7:431–35. 10.1016/S1470-2045(06)70695-7 16648048

[7] Hassler DM, Zeitlin C, Wimmer-Schweingruber RF, Ehresmann B, Rafkin S, Eigenbrode JL, Brinza DE, Weigle G, Böttcher S, Böhm E, Burmeister S, Guo J, Köhler J, et al, and MSL Science Team. Mars’ surface radiation environment measured with the Mars Science Laboratory’s Curiosity rover. Science. 2014; 343:1244797. 10.1126/science.1244797 24324275

[8] Zeitlin C, Hassler DM, Cucinotta FA, Ehresmann B, Wimmer-Schweingruber RF, Brinza DE, Kang S, Weigle G, Böttcher S, Böhm E, Burmeister S, Guo J, Köhler J, et al. Measurements of energetic particle radiation in transit to Mars on the Mars Science Laboratory. Science. 2013; 340:1080–84. 10.1126/science.1235989 23723233

[9] Schimmerling W. The space radiation environment: an introduction. https://threejscnasagov/concepts/SpaceRadiationEnvironpdf. 2011

[10] Durante M. New challenges in high-energy particle radiobiology. Br J Radiol. 2014; 87:20130626. 10.1259/bjr.20130626 24198199 PMC4064605

[11] Newhauser WD, Durante M. Assessing the risk of second malignancies after modern radiotherapy. Nat Rev Cancer. 2011; 11:438–48. 10.1038/nrc3069 21593785 PMC4101897

[12] Schulz-Ertner D, Jäkel O, Schlegel W. Radiation therapy with charged particles. Semin Radiat Oncol. 2006; 16:249–59. 10.1016/j.semradonc.2006.04.008 17010908

[13] Ishikawa H, Katoh H, Kaminuma T, Kawamura H, Ito K, Matsui H, Hirato J, Shimizu N, Takezawa Y, Tsuji H, Suzuki K, Ohno T, Nakano T, and Group for Genitourinary Tumors at Gunma Heavy Ion Medical Center. Carbon-ion Radiotherapy for Prostate Cancer: Analysis of Morbidities and Change in Health-related Quality of Life. Anticancer Res. 2015; 35:5559–66. 26408726

[14] Jäkel O, Karger CP, Debus J. The future of heavy ion radiotherapy. Med Phys. 2008; 35:5653–63. 10.1118/1.3002307 19175122

[15] Datta K, Suman S, Kallakury BV, Fornace AJ Jr. Exposure to heavy ion radiation induces persistent oxidative stress in mouse intestine. PLoS One. 2012; 7:e42224. 10.1371/journal.pone.0042224 22936983 PMC3427298

[16] Datta K, Suman S, Fornace AJ Jr. Radiation persistently promoted oxidative stress, activated mTOR via PI3K/Akt, and downregulated autophagy pathway in mouse intestine. Int J Biochem Cell Biol. 2014; 57:167–76. 10.1016/j.biocel.2014.10.022 25449263 PMC4363107

[17] Suman S, Kumar S, Fornace AJ, Datta K. Space radiation exposure persistently increased leptin and IGF1 in serum and activated leptin-IGF1 signaling axis in mouse intestine. Sci Rep. 2016; 6:31853. 10.1038/srep31853 27558773 PMC4997262

[18] Suman S, Kumar S, Fornace AJ Jr, Datta K. The effect of carbon irradiation is associated with greater oxidative stress in mouse intestine and colon relative to γ-rays. Free Radic Res. 2018; 52:556–67. 10.1080/10715762.2018.1452204 29544379

[19] Datta K, Suman S, Kallakury BV, Fornace AJ Jr. Heavy ion radiation exposure triggered higher intestinal tumor frequency and greater β-catenin activation than γ radiation in APC(Min/+) mice. PLoS One. 2013; 8:e59295. 10.1371/journal.pone.0059295 23555653 PMC3605451

[20] Suman S, Moon BH, Thakor H, Fornace AJ Jr, Datta K. Wip1 abrogation decreases intestinal tumor frequency in APC(Min/+) mice irrespective of radiation quality. Radiat Res. 2014; 182:345–49. 10.1667/RR13770.1 25117622 PMC5317043

[21] Suman S, Kumar S, Moon BH, Fornace AJ Jr, Datta K. Low and high dose rate heavy ion radiation-induced intestinal and colonic tumorigenesis in APC^1638N/+^ mice. Life Sci Space Res (Amst). 2017; 13:45–50. 10.1016/j.lssr.2017.04.003 28554509

[22] Metcalfe C, Kljavin NM, Ybarra R, de Sauvage FJ. Lgr5+ stem cells are indispensable for radiation-induced intestinal regeneration. Cell Stem Cell. 2014; 14:149–59. 10.1016/j.stem.2013.11.008 24332836

[23] Minafra L, Bravatà V, Cammarata FP, Di Maggio FM, Forte GI. SASPects of Radiation Induced Senescence. Ann Radiat Ther Oncol. 2017; 1:1006.

[24] Liao EC, Hsu YT, Chuah QY, Lee YJ, Hu JY, Huang TC, Yang PM, Chiu SJ. Radiation induces senescence and a bystander effect through metabolic alterations. Cell Death Dis. 2014; 5:e1255. 10.1038/cddis.2014.220 24853433 PMC4047910

[25] Li J, Poi MJ, Tsai MD. Regulatory mechanisms of tumor suppressor P16(INK4A) and their relevance to cancer. Biochemistry. 2011; 50:5566–82. 10.1021/bi200642e 21619050 PMC3127263

[26] Nilsson K, Landberg G. Subcellular localization, modification and protein complex formation of the cdk-inhibitor p16 in Rb-functional and Rb-inactivated tumor cells. Int J Cancer. 2006; 118:1120–25. 10.1002/ijc.21466 16161044

[27] Asada M, Yamada T, Ichijo H, Delia D, Miyazono K, Fukumuro K, Mizutani S. Apoptosis inhibitory activity of cytoplasmic p21(Cip1/WAF1) in monocytic differentiation. EMBO J. 1999; 18:1223–34. 10.1093/emboj/18.5.1223 10064589 PMC1171213

[28] Felisiak-Golabek A, Dansonka-Mieszkowska A, Rzepecka IK, Szafron L, Kwiatkowska E, Konopka B, Podgorska A, Rembiszewska A, Kupryjanczyk J. p19(INK4d) mRNA and protein expression as new prognostic factors in ovarian cancer patients. Cancer Biol Ther. 2013; 14:973–81. 10.4161/cbt.25966 24022213 PMC3926894

[29] Marazita MC, Ogara MF, Sonzogni SV, Martí M, Dusetti NJ, Pignataro OP, Cánepa ET. CDK2 and PKA mediated-sequential phosphorylation is critical for p19INK4d function in the DNA damage response. PLoS One. 2012; 7:e35638. 10.1371/journal.pone.0035638 22558186 PMC3338453

[30] Wagner J, Damaschke N, Yang B, Truong M, Guenther C, McCormick J, Huang W, Jarrard D. Overexpression of the novel senescence marker β-galactosidase (GLB1) in prostate cancer predicts reduced PSA recurrence. PLoS One. 2015; 10:e0124366. 10.1371/journal.pone.0124366 25876105 PMC4398352

[31] Li M, You L, Xue J, Lu Y. Ionizing Radiation-Induced Cellular Senescence in Normal, Non-transformed Cells and the Involved DNA Damage Response: A Mini Review. Front Pharmacol. 2018; 9:522. 10.3389/fphar.2018.00522 29872395 PMC5972185

[32] Barker N, van de Wetering M, Clevers H. The intestinal stem cell. Genes Dev. 2008; 22:1856–64. 10.1101/gad.1674008 18628392 PMC2735277

[33] Kim CK, Yang VW, Bialkowska AB. The Role of Intestinal Stem Cells in Epithelial Regeneration Following Radiation-Induced Gut Injury. Curr Stem Cell Rep. 2017; 3:320–32. 10.1007/s40778-017-0103-7 29497599 PMC5818549

[34] Schieber M, Chandel NS. ROS function in redox signaling and oxidative stress. Curr Biol. 2014; 24:R453–62. 10.1016/j.cub.2014.03.034 24845678 PMC4055301

[35] Bologna-Molina R, Mosqueda-Taylor A, Molina-Frechero N, Mori-Estevez AD, Sánchez-Acuña G. Comparison of the value of PCNA and Ki-67 as markers of cell proliferation in ameloblastic tumors. Med Oral Patol Oral Cir Bucal. 2013; 18:e174–79. 10.4317/medoral.18573 23229269 PMC3613329

[36] Connolly KM, Bogdanffy MS. Evaluation of proliferating cell nuclear antigen (PCNA) as an endogenous marker of cell proliferation in rat liver: a dual-stain comparison with 5-bromo-2′-deoxyuridine. J Histochem Cytochem. 1993; 41:1–6. 10.1177/41.1.7678022 7678022

[37] Schönenberger F, Deutzmann A, Ferrando-May E, Merhof D. Discrimination of cell cycle phases in PCNA-immunolabeled cells. BMC Bioinformatics. 2015; 16:180. 10.1186/s12859-015-0618-9 26022740 PMC4448323

[38] Chen JH, Hales CN, Ozanne SE. DNA damage, cellular senescence and organismal ageing: causal or correlative? Nucleic Acids Res. 2007; 35:7417–28. 10.1093/nar/gkm681 17913751 PMC2190714

[39] Campisi J. Aging, cellular senescence, and cancer. Annu Rev Physiol. 2013; 75:685–705. 10.1146/annurev-physiol-030212-183653 23140366 PMC4166529

[40] Bedir R, Güçer H, Şehitoğlu İ, Yurdakul C, Bağcı P, Üstüner P. The Role of p16, p21, p27, p53 and Ki-67 Expression in the Differential Diagnosis of Cutaneous Squamous Cell Carcinomas and Keratoacanthomas: An Immunohistochemical Study. Balkan Med J. 2016; 33:121–27. 10.5152/balkanmedj.2016.16442 27403379 PMC4924954

[41] Raderschall E, Bazarov A, Cao J, Lurz R, Smith A, Mann W, Ropers HH, Sedivy JM, Golub EI, Fritz E, Haaf T. Formation of higher-order nuclear Rad51 structures is functionally linked to p21 expression and protection from DNA damage-induced apoptosis. J Cell Sci. 2002; 115:153–64. 11801733 10.1242/jcs.115.1.153

[42] Koh SS, Cassarino DS. Immunohistochemical Expression of p16 in Melanocytic Lesions: An Updated Review and Meta-analysis. Arch Pathol Lab Med. 2018; 142:815–28. 10.5858/arpa.2017-0435-RA 29939777

[43] Solomon C, Louw M, van Aardt M, Dreyer G. p16 and Ki-67 immunohistochemical staining reduces inter- and intra-observer variability in the grading of cervical squamous intraepithelial lesions of South African women. Southern African Journal of Gynaecological Oncology. 2017; 9:25–29. 10.1080/20742835.2017.1370841

[44] Coskun V, Luskin MB. The expression pattern of the cell cycle inhibitor p19(INK4d) by progenitor cells of the rat embryonic telencephalon and neonatal anterior subventricular zone. J Neurosci. 2001; 21:3092–103. 10.1523/JNEUROSCI.21-09-03092.2001 11312294 PMC4211624

[45] Barker N, van Es JH, Kuipers J, Kujala P, van den Born M, Cozijnsen M, Haegebarth A, Korving J, Begthel H, Peters PJ, Clevers H. Identification of stem cells in small intestine and colon by marker gene Lgr5. Nature. 2007; 449:1003–07. 10.1038/nature06196 17934449

[46] Baulch JE, Craver BM, Tran KK, Yu L, Chmielewski N, Allen BD, Limoli CL. Persistent oxidative stress in human neural stem cells exposed to low fluences of charged particles. Redox Biol. 2015; 5:24–32. 10.1016/j.redox.2015.03.001 25800120 PMC4371546

[47] Limoli CL, Giedzinski E, Baure J, Rola R, Fike JR. Redox changes induced in hippocampal precursor cells by heavy ion irradiation. Radiat Environ Biophys. 2007; 46:167–72. 10.1007/s00411-006-0077-9 17103219

[48] Suman S, Rodriguez OC, Winters TA, Fornace AJ Jr, Albanese C, Datta K. Therapeutic and space radiation exposure of mouse brain causes impaired DNA repair response and premature senescence by chronic oxidant production. Aging (Albany NY). 2013; 5:607–22. 10.18632/aging.100587 23928451 PMC3796214

[49] Bigarella CL, Liang R, Ghaffari S. Stem cells and the impact of ROS signaling. Development. 2014; 141:4206–18. 10.1242/dev.107086 25371358 PMC4302918

[50] Hochmuth CE, Biteau B, Bohmann D, Jasper H. Redox regulation by Keap1 and Nrf2 controls intestinal stem cell proliferation in Drosophila. Cell Stem Cell. 2011; 8:188–99. 10.1016/j.stem.2010.12.006 21295275 PMC3035938

[51] Kiraly O, Gong G, Olipitz W, Muthupalani S, Engelward BP. Inflammation-induced cell proliferation potentiates DNA damage-induced mutations in vivo. PLoS Genet. 2015; 11:e1004901. 10.1371/journal.pgen.1004901 25647331 PMC4372043

[52] Berger E, Rath E, Yuan D, Waldschmitt N, Khaloian S, Allgäuer M, Staszewski O, Lobner EM, Schöttl T, Giesbertz P, Coleman OI, Prinz M, Weber A, et al. Mitochondrial function controls intestinal epithelial stemness and proliferation. Nat Commun. 2016; 7:13171. 10.1038/ncomms13171 27786175 PMC5080445

[53] Chen F, Liu Y, Wong NK, Xiao J, So KF. Oxidative Stress in Stem Cell Aging. Cell Transplant. 2017; 26:1483–95. 10.1177/0963689717735407 29113471 PMC5680960

[54] Richardson RB. Ionizing radiation and aging: rejuvenating an old idea. Aging (Albany NY). 2009; 1:887–902. 10.18632/aging.100081 20157573 PMC2815743

[55] Tchkonia T, Zhu Y, van Deursen J, Campisi J, Kirkland JL. Cellular senescence and the senescent secretory phenotype: therapeutic opportunities. J Clin Invest. 2013; 123:966–72. 10.1172/JCI64098 23454759 PMC3582125

[56] Dreesen O, Chojnowski A, Ong PF, Zhao TY, Common JE, Lunny D, Lane EB, Lee SJ, Vardy LA, Stewart CL, Colman A. Lamin B1 fluctuations have differential effects on cellular proliferation and senescence. J Cell Biol. 2013; 200:605–17. 10.1083/jcb.201206121 23439683 PMC3587829

[57] Freund A, Laberge RM, Demaria M, Campisi J. Lamin B1 loss is a senescence-associated biomarker. Mol Biol Cell. 2012; 23:2066–75. 10.1091/mbc.e11-10-0884 22496421 PMC3364172

[58] Cucinotta FA, Wu H, Shavers MR, George K. Radiation dosimetry and biophysical models of space radiation effects. Gravit Space Biol Bull. 2003; 16:11–18. 12959127

[59] Li M, Gonon G, Buonanno M, Autsavapromporn N, de Toledo SM, Pain D, Azzam EI. Health risks of space exploration: targeted and nontargeted oxidative injury by high-charge and high-energy particles. Antioxid Redox Signal. 2014; 20:1501–23. 10.1089/ars.2013.5649 24111926 PMC3936510

[60] Coppé JP, Desprez PY, Krtolica A, Campisi J. The senescence-associated secretory phenotype: the dark side of tumor suppression. Annu Rev Pathol. 2010; 5:99–118. 10.1146/annurev-pathol-121808-102144 20078217 PMC4166495

[61] Ungvari Z, Podlutsky A, Sosnowska D, Tucsek Z, Toth P, Deak F, Gautam T, Csiszar A, Sonntag WE. Ionizing radiation promotes the acquisition of a senescence-associated secretory phenotype and impairs angiogenic capacity in cerebromicrovascular endothelial cells: role of increased DNA damage and decreased DNA repair capacity in microvascular radiosensitivity. J Gerontol A Biol Sci Med Sci. 2013; 68:1443–57. 10.1093/gerona/glt057 23689827 PMC3814240

[62] Kim SB, Bozeman RG, Kaisani A, Kim W, Zhang L, Richardson JA, Wright WE, Shay JW. Radiation promotes colorectal cancer initiation and progression by inducing senescence-associated inflammatory responses. Oncogene. 2016; 35:3365–75. 10.1038/onc.2015.395 26477319 PMC4837107

[63] Richmond CA, Rickner H, Shah MS, Ediger T, Deary L, Zhou F, Tovaglieri A, Carlone DL, Breault DT. JAK/STAT-1 Signaling Is Required for Reserve Intestinal Stem Cell Activation during Intestinal Regeneration Following Acute Inflammation. Stem Cell Reports. 2018; 10:17–26. 10.1016/j.stemcr.2017.11.015 29276155 PMC5768934

